# *Drosophila* Smad2 Opposes Mad Signaling during Wing Vein Development

**DOI:** 10.1371/journal.pone.0010383

**Published:** 2010-04-28

**Authors:** Veronika Sander, Edward Eivers, Renee H. Choi, Edward M. De Robertis

**Affiliations:** Howard Hughes Medical Institute, Department of Biological Chemistry, University of California Los Angeles, Los Angeles, California, United States of America; Columbia University, United States of America

## Abstract

In the vertebrates, the BMP/Smad1 and TGF-β/Smad2 signaling pathways execute antagonistic functions in different contexts of development. The differentiation of specific structures results from the balance between these two pathways. For example, the gastrula organizer/node of the vertebrates requires a region of low Smad1 and high Smad2 signaling. In *Drosophila*, Mad regulates tissue determination and growth in the wing, but the function of dSmad2 in wing patterning is largely unknown. In this study, we used an RNAi loss-of-function approach to investigate dSmad2 signaling during wing development. RNAi-mediated knockdown of dSmad2 caused formation of extra vein tissue, with phenotypes similar to those seen in Dpp/Mad gain-of-function. Clonal analyses revealed that the normal function of dSmad2 is to inhibit the response of wing intervein cells to the extracellular Dpp morphogen gradient that specifies vein formation, as measured by expression of the activated phospho-Mad protein. The effect of dSmad2 depletion in promoting vein differentiation was dependent on Medea, the co-factor shared by Mad and dSmad2. Furthermore, double RNAi experiments showed that Mad is epistatic to dSmad2. In other words, depletion of Smad2 had no effect in Mad-deficient wings. Our results demonstrate a novel role for dSmad2 in opposing Mad-mediated vein formation in the wing. We propose that the main function of dActivin/dSmad2 in *Drosophila* wing development is to antagonize Dpp/Mad signaling. Possible molecular mechanisms for the opposition between dSmad2 and Mad signaling are discussed.

## Introduction

Signaling by the transforming growth factor-β (TGF-β) superfamily of ligands is important for proliferation, differentiation, and cell fate determination during embryonic development and tissue homeostasis in the adult. This group of ligands can be divided into two broad signaling families, the Bone Morphogenetic Protein (BMP) and the TGF-β/Activin pathways. Their signal transduction pathways are highly conserved in animal species, and homologues of most components have been described from nematodes to humans. A group of transcription factors known as R-Smads (Receptor-Smads) transduce the intracellular signal of each pathway.

In the vertebrates, the BMP pathway signals through Smad1/5/8, while Smad2/3 relay signals of the TGF-β/Activin pathway [1]. BMP ligands bind to and activate BMP receptors (BMPR, type I and II), causing Smad1/5/8 phosphorylation at its two C-terminal serines (SVS). Phosphorylated Smad1^cter^ then binds to Smad4 (co-Smad) and this complex translocates and accumulates in the nucleus, activating BMP-responsive genes. The pSmad1^cter^ signal is fine-tuned by a number of inhibitory phosphorylations at Mitogen Activated Protein Kinase (MAPK) and Glycogen Synthase Kinase 3 (GSK3) sites in the linker/middle domain of the protein. Linker phosphorylation results in the rapid termination of the BMP/Smad1 signal by a process of polyubiquitination followed by proteasomal degradation in the centrosomal region of the cell [2]–[8]. The reverse effect is mediated by PP2A (Protein Phosphatase 2A), which has been shown to be the first stimulatory phosphatase of the BMP pathway. PP2A preferentially dephosphorylates the Smad1 linker region, leading to prolonged BMP signaling [9]. In *Drosophila*, BMP signaling is mediated by the secreted ligands Decapentaplegic (Dpp), Screw (Scw) and Glass bottom boat (Gbb), the type I receptors Thickveins (Tkv) and Saxophone (Sax), the type II receptors Punt and Wishful thinking (Wit) the Smad1/5/8 homolog Mothers against Dpp (Mad), and the Smad4 homolog Medea [10]–[12].

TGF-β/Activin signals are transduced in a similar fashion as the BMP branch, via binding to type I and II receptors, C-terminal phosphorylation of Smad2/3 (at SMS or SVS sites), followed by complex formation with Smad4 and transcriptional activation of target genes in the nucleus [1]. Several studies have addressed the function of the Activin pathway in *Drosophila* [13]–[18]. The secreted ligands *Drosophila* Activin (dAct) and Dawdle (Daw) have been shown to signal through the type I receptor Baboon (Babo) [13], [18]. dSmad2 was first described by Henderson and Andrew and named Smox, for Smad on chromosome X [19]. Sequence alignments revealed high similarity of dSmad2 to vertebrate Smad2 and Smad3. Functionally, it appears that dSmad2 is more similar to Smad3, due to the lack of a 30 amino acids insertion in the MH1 domain present in some isoforms of vertebrate Smad2 that prevent binding to DNA [11], [13], [14]. It should be noted that the dAct/dSmad2 signal transduction pathway shares three components with the Dpp/Mad branch: the type II receptors Punt and Wit, and the co-Smad Medea [14], [17].

In several situations during embryonic development, Smad1 and Smad2 signals have been shown to function antagonistically. For example, in the *Xenopus* gastrula two morphogens have been identified: BMP4 and TGF-β/Nodal. These growth factors establish a bidirectional antagonistic embryonic field, which is interpreted by intracellular Smad1 and Smad2 signals [20]. The antagonism takes place at different levels. Extracellularly, Nodal/Smad2 induce the transcription of anti-BMPs, such as Chordin and Noggin, which inhibit BMP4 by binding to it [21]. Intracellularly, Smad1 inhibits activation of Smad2 transcriptional targets, and Smad2 activation inhibits Smad1 target genes [20], [22]–[25]. In this way, mutually exclusive regions of high Smad1 or Smad2 signaling are established on opposite sides of the early embryo. At the level of tissue-specific Smad-induced transcription factors, this antagonism is illustrated by the opposing functions of the homeobox genes *Vent1/2*, induced by Smad1 on the ventral side, and *Goosecoid*, a target gene of Smad2 on the dorsal side, during mesoderm patterning and self-regulation of the early frog embryo [26]. Another example of the importance of the antagonism between Smad1 and Smad2 was recently reported by Yamamoto and co-workers [27]. The authors demonstrated that development of the distal visceral endoderm, which determines the orientation of the anterior-posterior axis in the mouse, requires the formation of a region where Smad2 signaling is high and Smad1 is absent.

The *Drosophila* wing provides an excellent model system to study intercellular signal regulation [28]. Dpp/Mad promotes vein differentiation during larval and pupal stages of development [29], [30], as well as wing growth [31]–[34]. Loss-of-function mutations at the ligand, receptor or transcription factor level of the Dpp signaling pathway have been shown to be required for vein formation and wing growth. Conversely, overexpression of Dpp, activated Tkv receptor or degradation-resistant Mad caused increased vein formation [6], [12], [35].

The role of dSmad2 in wing development is much less understood in *Drosophila*. It has been reported that dSmad2 may play a modest role in the proliferation of wing cells, without affecting patterning and gene expression [13], [17]. These studies were performed by overexpression of activated dSmad2 (phospho-mimicking mutations of the C-terminal serines, dSmad2-DMD) [17], or, in a more indirect approach, using a constitutively-active and mutant version of Babo receptor [13]. As for dSmad2 mutations, there is only one dSmad2/Smox allele available, a missense mutation that has not been studied in the wing [15].

The present study was initiated to determine the role of dSmad2 during wing development through a loss-of-function approach using the powerful RNAi technology that has recently become available [36], [37]. To our surprise, we found that dSmad2 serves mainly to oppose signaling through Mad in the developing wing. Depletion of dSmad2 in wing imaginal discs caused increased vein tissue development. This was the opposite of Mad knockdown, which inhibited vein formation. Analysis of dSmad2 RNAi clones revealed an increase in nuclear phospho-Mad levels in pupal wings during vein formation. In double RNAi depletion experiments, Mad was epistatic to dSmad2, showing that the dSmad2 loss-of-function phenotypes arose through increased Mad activity. We discuss possible molecular mechanisms that might explain the inhibitory effect of dSmad2 on Mad.

## Results

### RNAi-mediated knockdown of dSmad2 increases vein formation in *Drosophila* wings

**Figure 1:**
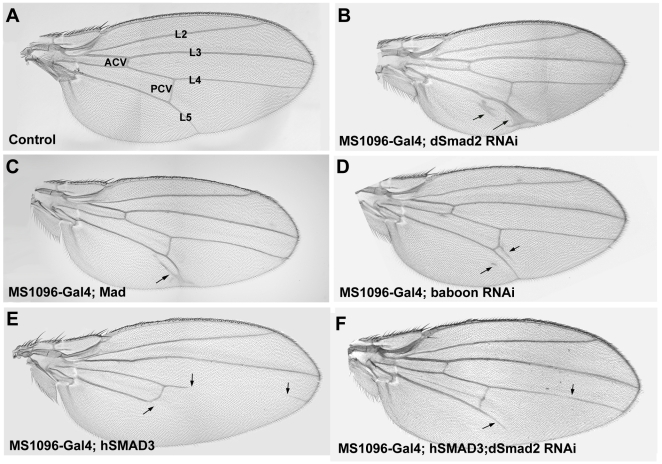
dSmad2 RNAi induces vein tissue differentiation. (A) Control wing showing the location of the longitudinal veins 2–5 (L2–L5); anterior crossvein (ACV); posterior crossvein (PCV). (B) dSmad2 RNAi driven by MS1096-Gal4 leads ectopic vein tissue formation in the vicinity of L5 (arrows). (C) A similar phenotype is observed in flies overexpressing Mad. (E) Overexpression of the dSmad2 homologue human Smad3 results in a partial loss of L4 and L5; an identical phenotype is seen in some Dpp partial loss-of-function mutants [48]. (F) human Smad3 rescues the dSmad2 RNAi phenotype (arrows), demonstrating dSmad2 RNAi specifically depletes dSmad2.

We investigated the function of dSmad2 in *Drosophila* wing development using UAS/RNAi transgenic flies from the Vienna Drosophila RNAi Center. Driving UAS-dSmad2 RNAi ubiquitously in the wing blade using the MS1096-Gal4 driver resulted in increased vein tissue formation along longitudinal vein 5 (L5), when compared to an adult wild-type wing (compare Figure 1A to 1B). This phenotype was similar to the overexpression of UAS-Mad wild-type (Figure 1C). The dSmad2 RNAi used in most of this study targeted a 400 bp nucleotide fragment corresponding to the linker domain and part of the MH2 region of dSmad2 (GD14609, Figure S1A in Supporting Information). A second independent RNAi line targeting the MH1 domain of dSmad2 (KK105687, Figure S1A) was driven under the same conditions, and also caused ectopic vein tissue along L5 (Figure S1A). The increase in vein tissue caused by dSmad2 RNAi was rescued to wild-type levels by co-expression of a human SMAD3 transgene (compare Figure 1B to 1F). Taken together, these findings indicate that the function of endogenous dSmad2 is to negatively regulate vein formation in the *Drosophila* wing.

Another indication that dSmad2 knockdown causes elevated Mad signaling was the appearance of ectopic sensory bristles in wing blade intervein tissues (Figure S2). Ectopic bristles are also observed when hyperactive Mad mutants are overexpressed in the fly wing [6]. We next investigated further the hypothesis that loss of dSmad2 phenocopies an increase in Dpp/Mad signaling.

### dSmad2 knockdown phenocopies Dpp overexpression in the wing disc

**Figure 2:**
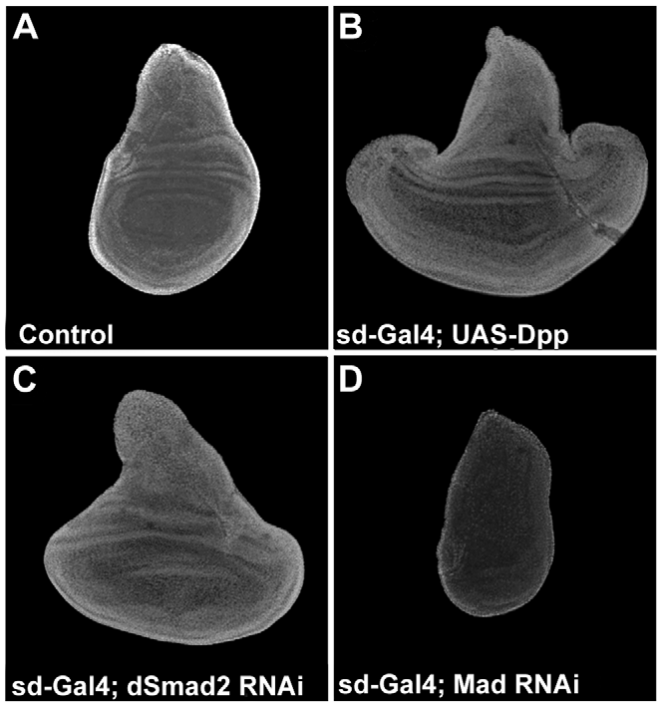
Knockdown of dSmad2 resembles Dpp overexpression in the wing disc. (A) Control imaginal wing disc at third instar stage, expressing Scalloped (Sd)-Gal4 throughout larval development of the wing. (B) Overexpression of Dpp causes overgrowth of the wing blade region of the disc. (C) dSmad2 RNAi phenocopies Dpp overexpression, expanding the wing blade region. (D) Conversely, downregulation of the Dpp pathway by Mad RNAi reduces the size of the wing disc.

Analysis of third instar wing imaginal discs overexpressing UAS-Dpp (driven by Scalloped-Gal4), dSmad2 RNAi or Mad RNAi revealed further phenotypic similarities between dSmad2 loss-of-function and Mad gain-of-function. Overexpression of Dpp (which activates Mad) or depletion of dSmad2 resulted in overgrowth of wing imaginal discs when compared to wild-type discs (Figure 2A–2C) [12]. Depletion of the Mad transcription factor using RNAi [6] caused inhibition of wing imaginal disc growth (Figure 2D). Overgrowth of wing discs by dSmad2 RNAi was prominent when driven by scalloped-Gal4, but weaker when driven by MS1096-Gal4; this may be explained by spatiotemporal differences in driver expression. The results indicate that while Dpp/Mad signaling promotes wing imaginal disc growth, endogenous dSmad2 signaling has the opposite effect.

### dSmad2 RNAi causes transformation of intervein into vein tissue

**Figure 3:**
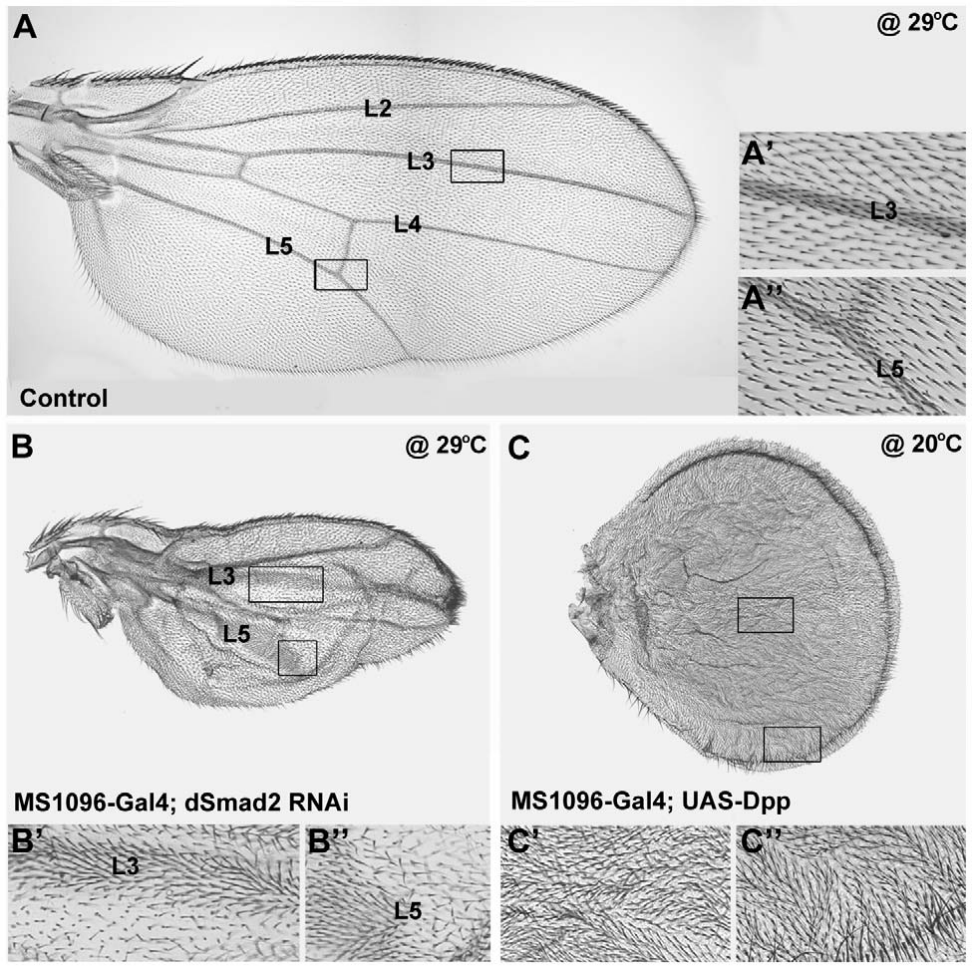
dSmad2 depletion leads to transformation of intervein into vein tissue. (A) Wild-type wing retains normal size and vein patterning when grown at 29°C. A’ and A” insets show higher magnification of L3 and L5 regions to illustrate the size differences between vein and intervein cells, each of which have a single hair (trichome) (B) Driving dSmad2 RNAi with MS1096-Gal4 at 29°C results in small wings due to increase in vein tissue mostly near L3 (B’) and L5 (B”). (C) Dpp-overexpressing flies display a similar wing phenotype to dSmad2 RNAi, although stronger. High levels of Dpp/Mad lead to an overproduction of vein, resulting in wings of reduced size that are composed entirely of vein tissue (C’ and C”). Flies overexpressing Dpp in the wing only survive to adult stages when grown at low temperature (20°C).

Increased size of the third instar wing imaginal disc does not necessarily generate enlarged adult wings. Mature vein cells are smaller and more densely packed than intervein cells, in addition to secreting more pigmented cuticle [29], [38]. In a situation in which more vein cells relative to intervein cells were differentiated, without increasing overall cell proliferation, the final size of the adult wing would be decreased. In addition, other processes such as blisters due to imperfect alignment of the two wing surfaces or increased apoptosis could contribute to a decrease in wing size. In case of dSmad2 knockdown (driven by MS1096-Gal4), we observed a smaller adult wing size accompanied by moderately increased vein formation at room temperature (Figure S1E). These effects became more severe when the flies were grown at a higher temperature (29°C) (Figure 3B). dSmad2-depleted wings were much smaller than control wings at this temperature, and displayed significantly wider L3 and L5 veins (compare Figure 3A to 3B). The smaller size and higher cell density of vein cells (each wing cell is marked by a single trichome) compared to neighboring intervein cells [29] is readily seen at higher magnifications (insets in Figure 3A’–3C”).

We compared the dSmad2 RNAi phenotype to gain-of-function of Dpp in the adult wing. Despite the massive overgrowth of the wing imaginal disc by overexpressing UAS-Dpp (Figure 2B), at 20°C (flies do not eclose at higher temperatures) the resulting adult wings were found to be significantly smaller than wild-type (Figure 3C). This size difference is explained by the transformation of the larger intervein cells into much smaller vein cells and higher cell density (Figure 3C’ and 3C”). The similarity of the phenotypes of dSmad2 loss-of-function and Dpp/Mad gain-of-function in imaginal discs (Figure 2) and in adult wings (Figure 3) suggested that an antagonism exists between the dSmad2 and Mad signaling pathways during *Drosophila* wing differentiation.

### dSmad2 RNAi clones have increased Mad^cter^ phosphorylation in vein tissue

**Figure 4:**
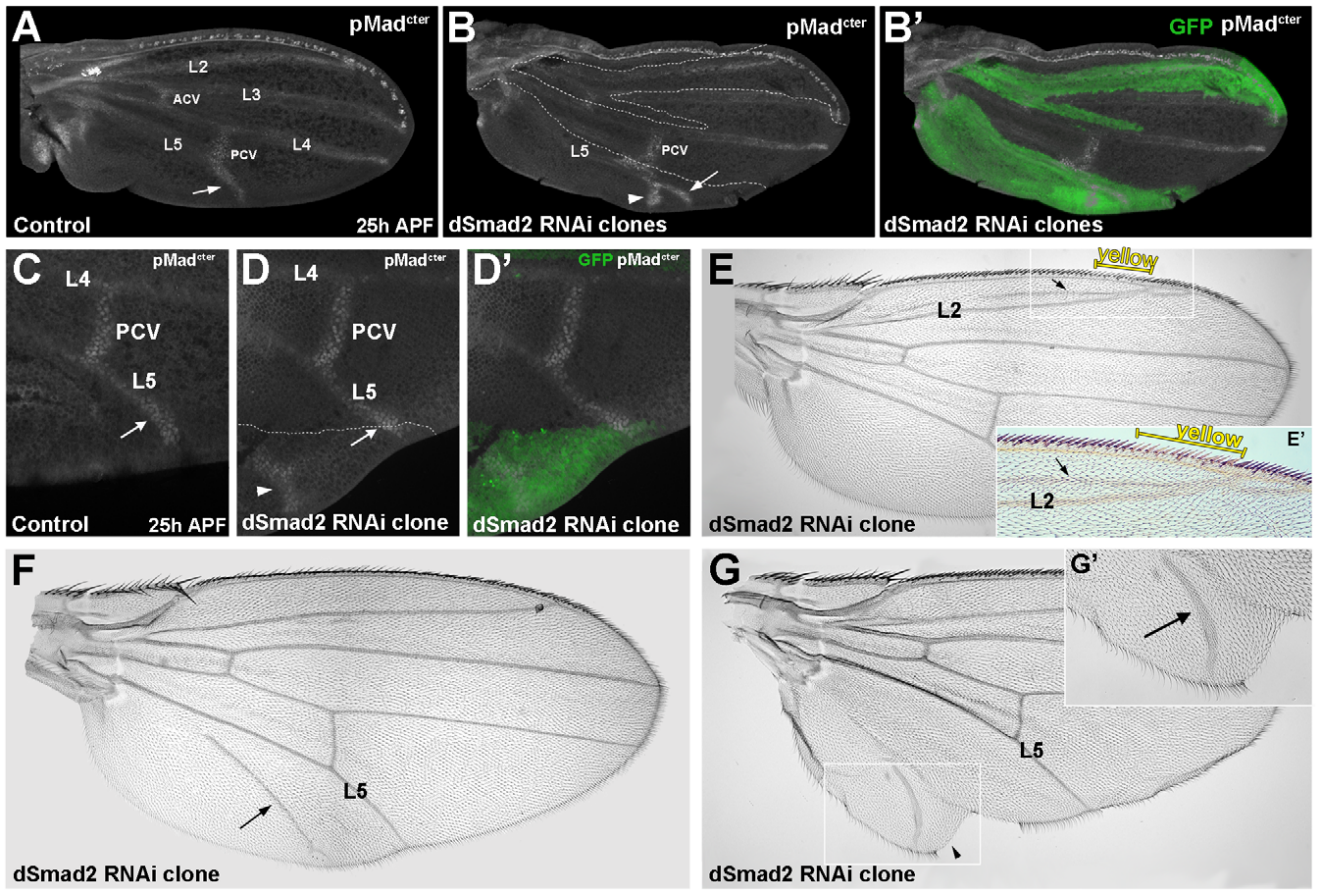
pMad is expressed in ectopic veins induced within dSmad2 RNAi clones. (A) Pupal wing 25 hrs after puparium formation (APF) stained with pMad^cter^ antibody. Nuclear pMad staining is visible in the crossveins and longitudinal veins, most prominently at the distal tips. (B and B’) dSmad2 RNAi clone marked with GFP induced an ectopic pMad^cter^ expressing vein (arrowhead) posterior to L5 (arrow). (C–D’) Similar result shown in an independent experiment. Note the outgrowth of the wing surrounding the ectopic vein (arrowhead in D). (E) dSmad2 clone marked by *yellow* (y+) in anterior margin bristles induced the formation of an ectopic vein between the anterior margin and L2. (F) Ectopic vein induced by dSmad2 clone in the posterior compartment (arrow). (G) dSmad2 clone inducing ectopic vein (arrow in G’) and intervein tissue (arrowhead in G’).

We next investigated the mechanism of the proposed antagonism between dSmad2 and Mad by clonal analysis. A *Drosophila* strain was constructed, in which dSmad2 RNAi flp-out clones were marked by GFP in pupal wings and by *yellow* (*y+*) bristles in adult wings [39]. Activation of Mad signaling was followed by immunostaining with anti-phospho-Mad^cter^ antibody [40], [41] on pupal wings. C-terminal phosphorylation of Mad marking Dpp pathway activity becomes visible around 22 hrs after puparium formation (APF) in the crossveins, and soon after in the longitudinal veins [41]. Figure 4A shows pMad^cter^ staining at 25 hrs APF in a wild-type pupal wing. Large clones of dSmad2 RNAi were obtained by a 1 hr heat-shock (37°C) 24 hrs after egg laying (AEL) (Figure 4B’, clones labeled by GFP).

We observed ectopic vein formation in dSmad2 RNAi clones predominantly in the posterior wing compartment (Figure 4B and 4B’). Importantly, regions of increased pMad^cter^ were observed (arrowheads in Figure 4B and 4D). These were not uniform throughout the clone, but instead appeared to correspond to regions of ectopic vein formation, for they displayed nuclear staining for pMad^cter^ at comparable levels to those of endogenous pMad along L5 (arrows in Figure 4A–4D). The simplest interpretation is that knockdown of dSmad2 causes increased Mad signaling by making dSmad2-depleted cells more sensitive to endogenous Dpp signals [38], although other mechanisms remain possible. Although GFP-positive clones were distributed evenly over the whole wing, we found ectopic vein formation primarily in the posterior wing compartment near L5 and, in fewer cases, near the anterior margin (Figure 4G and 4G’). These regional effects may be due to the graded activity of endogenous pMad^cter^, which is normally lower far from the source of Dpp in the anterior-posterior compartment boundary [34], [42].

The increase in Mad signaling presumably explains the ectopic vein phenotypes seen in dSmad2 RNAi clones in adult wings (Figure 4E–4G). Although the clones are no longer visible with GFP in the adult wing blade, the region of the clones could still be identified in the anterior compartment by *yellow* bristles along the wing margin. Figure 4E shows a clone in the anterior margin marked by *yellow* bristles, with a nearby ectopic longitudinal vein originating between the anterior margin and L2 (arrow). In addition to ectopic veins, increased growth of intervein tissue in the vicinity of the extra vein was also observed (Figure 4G, arrowhead). These growths are indicative of ectopic wing formation, which can be caused by increased Dpp signaling [32], [34]. Taken together, the studies in clones suggest that dSmad2 RNAi causes excessive vein formation by increasing pMad^cter^ signaling.

### dSmad2 requires Medea for its inhibitory effect on vein formation

**Figure 5:**
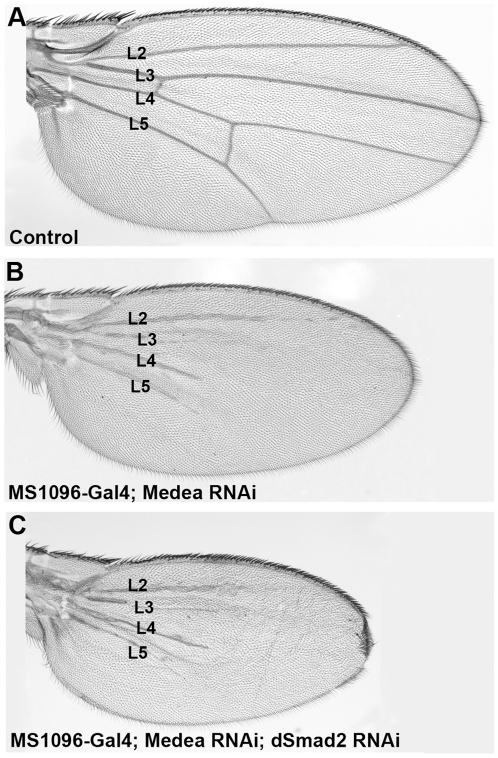
Medea is required for the effects of dSmad2 on vein formation. (A and B) Medea RNAi causes marked reduction of veins in the distal part of the wing. (C) Simultaneous depletion of Medea and dSmad2 affects vein formation in a similar way as knockdown of Medea alone. Note that the wing blade is smaller. The remaining wing veins are numbered.

The loss-of-function studies described above indicate that dSmad2 opposes Mad signaling during vein differentiation in the fly wing. The mechanism for this opposition, however, remains unclear. Both Mad and dSmad2 form a complex with the co-Smad Medea before binding to DNA and activating target gene expression [13], [43], [44]. To test whether Medea is required for the inhibitory effect of dSmad2 on vein formation, we analyzed the phenotype of depleting both genes in a combinational RNAi knockdown approach. We used the UAS-Medea RNAi construct KK106767 that targets nucleotides 1434 to 1883. Driving Medea RNAi alone in the wing blade resulted in smaller wings with great losses of vein tissue (Figure 5B). The residual vein tissue fails to reach the distal margin, although the five normal veins can stil be recognized. A second Medea RNAi construct had similar effects (not shown). Depletion of dSmad2 results in increased venation (Figure 1B). Simultaneous depletion of dSmad2 and Medea caused vein tissue loss and a further reduction in size (Figure 5C). This result indicates that Medea is required for the increased vein formation caused by knockdown of dSmad2. The further reduction of wing size when dSmad2 is depleted in combination with Medea is also of interest, because it takes place in the almost complete absence of vein tissue (Figure 5B and 5C). Thus, this decrease in size cannot be explained by changes in the ratio of intervein/vein cells, and suggests that dSmad2 also has a growth-promoting function in the wing, which is independent of its effects on Mad/Medea signaling.

### Mad is epistatic to dSmad2

**Figure 6:**
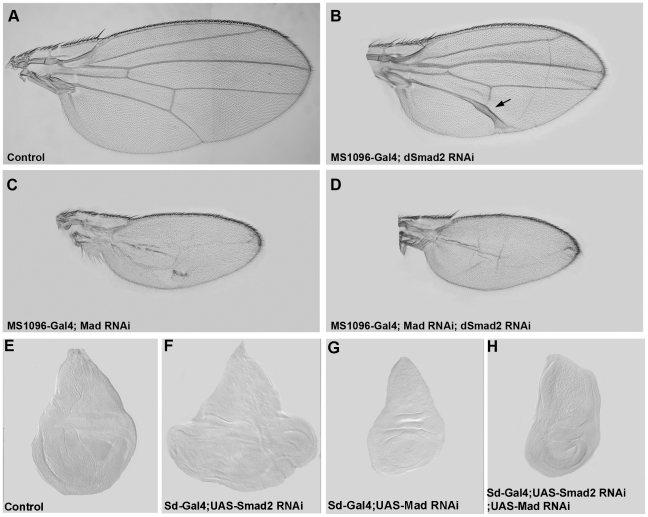
Mad is epistatic to dSmad2. (A and B) MS1096-Gal4 driving dSmad2 RNAi causes an increase of vein tissue in L5. (C) Depletion of Mad by RNAi results in small wings lacking veins. (D) Mad;dSmad2 double RNAi wings show identical phenotypes to Mad depletion alone – complete loss of veins and a small wing blade – showing that Mad is epistatic to dSmad2. (E) Wild type third instar wing imaginal disc. (F) Wing discs become enlarged when dSmad2 is depleted using RNAi driven by sd-Gal4. (G) Mad depletion reduces wing disc size. (H) Mad is required for the enlargement of wing discs caused by dSmad2 depletion, compare panels F and H.

To further investigate the epistatic relationship between dSmad2 and Mad in wing development, we compared single RNAi phenotypes for both genes to the double depletion situation. The UAS-Mad RNAi construct used targeted the N-terminal domain (nucleotides 226 to 807) [6]. As described above, dSmad2 RNAi driven in the wing blade formed extra vein tissue around L5 (Figure 6B). RNAi-mediated depletion of Mad resulted in the complete loss of veins and a strong reduction of the size of the wing blade (Figure 6C). The exact same phenotype was obtained in the double dSmad2;Mad RNAi cross, indicating that Mad is epistatic to dSmad2 (Figure 6D). Moreover, Mad was required for the overgrowth in wing disc tissue caused by *Drosophila* Smad2 RNAi using the sd-Gal4 driver (Figure 6E–6H). We conclude that the effects of dSmad2 depletion on adult wing vein formation and larval wing disc size require Mad function.

## Discussion

We investigated the role of dSmad2 in *Drosophila* wing development. Using RNAi lines, which specifically target and knockdown dSmad2, Mad and Medea, we found a novel role of dSmad2 in suppressing vein formation in a Mad/Medea-dependent way. Depletion of dSmad2 generated Mad gain-of-function phenotypes, and Mad was required for these dSmad2 loss-of-function phenotypes. We conclude that dSmad2 functions in wing development to inhibit Dpp/Mad signaling.

### dSmad2 opposes Mad in wing vein formation

When dSmad2 RNAi was driven throughout the wing blade, it caused an increase of vein tissue (Figure 1B). This phenotype resembles elevated Dpp/Mad signaling, which is known to promote vein differentiation in the wing [45]–[47]. Misexpression of activators of the pathway, such as Dpp, constitutively-active Tkv receptor, and wild-type or stabilized Mad all induce increased vein tissue to varying degrees [6], [35]. The opposite phenotype is seen in loss-of-function situations of the Dpp/Mad pathway. For example, both Medea RNAi and Mad RNAi cause loss of vein tissue (Figures 5B and 6C) [6]. Overexpression of hSMAD3, the vertebrate Smad most similar to dSmad2, precisely phenocopies the partial (adult viable) dpp^s6^/dpp^hr4^ loss-of-function phenotype, which displays truncations in L4 and L5 (compare Figure 1E to Figure 4 in [48]). In addition to Dpp/Mad, other signaling pathways are important in vein development in *Drosophila*. For example, EGF/MAPK, Notch and Hedgehog signaling are important in wing patterning, and disc growth requires Wg signaling and JAK/STAT activity [38].

We found that low dSmad2 signaling and high Dpp/Mad signaling result in similar wing vein phenotypes, indicating that dSmad2 antagonizes Mad in wing tissue determination. The increase in vein tissue upon dSmad2 knockdown is typically manifested around L5 in the posterior compartment of the wing (Figure 3B). Loss of vein tissue in hSMAD3-expressing wings occurs in the distal parts of L5 and, to a lesser extent, L4. These site-specific effects on vein formation might be a consequence of the expression of endogenous Dpp in a stripe along the anterior-posterior border in the imaginal disc. Dpp phosphorylates Mad, which forms a gradient that is highest near the source of Dpp, and low at the lateral areas of the wing disc. The pMad gradient is steeper in the posterior compartment [42]. Since L5 originates in the steeper posterior half in an area of low pMad, this region may be particularly susceptible to changes in cell sensitivity to the Dpp morphogen gradient.

Clonal analysis supported a role of endogenous dSmad2 in inhibiting Mad-mediated vein formation. dSmad2 RNAi clones showed higher levels of nuclear pMad^cter^ in ectopic vein-like regions of the pupal wing (Figure 4). Not all cells in the dSmad2 RNAi clones had higher pMad^cter^ phosphorylation. Veins and crossveins are induced by the flow of Dpp/Gbb morphogens in the developing wing field [30], [38]. In addition to the formation of ectopic vein tissue, some dSmad2 RNAi clones on the posterior margin caused wing outgrowths (Figure 4G). This effect is most likely due to increased cellular sensitivity to Dpp/Mad signaling, for it is reminiscent of Dpp clones that act as long-range wing organizers, inducing wing outgrowths and duplications [31]–[34].

### Mad is required for dSmad2 vein phenotypes

Epistasis experiments were performed using *Drosophila* lines expressing single or double RNAi transgenes in order to study the cross-regulation between dSmad2, Mad and Medea. Mad RNAi depletion caused complete loss of veins and a strong reduction in wing size. The exact same phenotype was obtained in the double Mad;dSmad2 knockdown (Figure 6). Similarly, the effects of Drosophila Smad2 knockdown on wing disc growth also required Mad (Figure 6E–6H). This shows that dSmad2 requires Mad in order to cause its principal phenotypes in wing development.

Wing pattern specification by Mad requires Medea [49]. RNAi-mediated knockdown of Medea caused a similar phenotype to that of Mad depletion, but was weaker. This may be due to a partial loss-of-function effect of the two Medea RNAi constructs tested in this study, or to Medea-independent functions of Mad. Medea loss-of-function mutations have been described to cause weaker phenotypes than Mad mutants [11], [43], [44]. When dSmad2 and Medea were depleted simultaneously, we found that Medea was required for vein induction by dSmad2 RNAi (Figure 5). We conclude from these epistatic experiments that both Mad and Medea, which normally bind to each other, are required for the dSmad2 knockdown vein phenotypes.

### How does dSmad2 oppose Mad?

**Figure 7:**
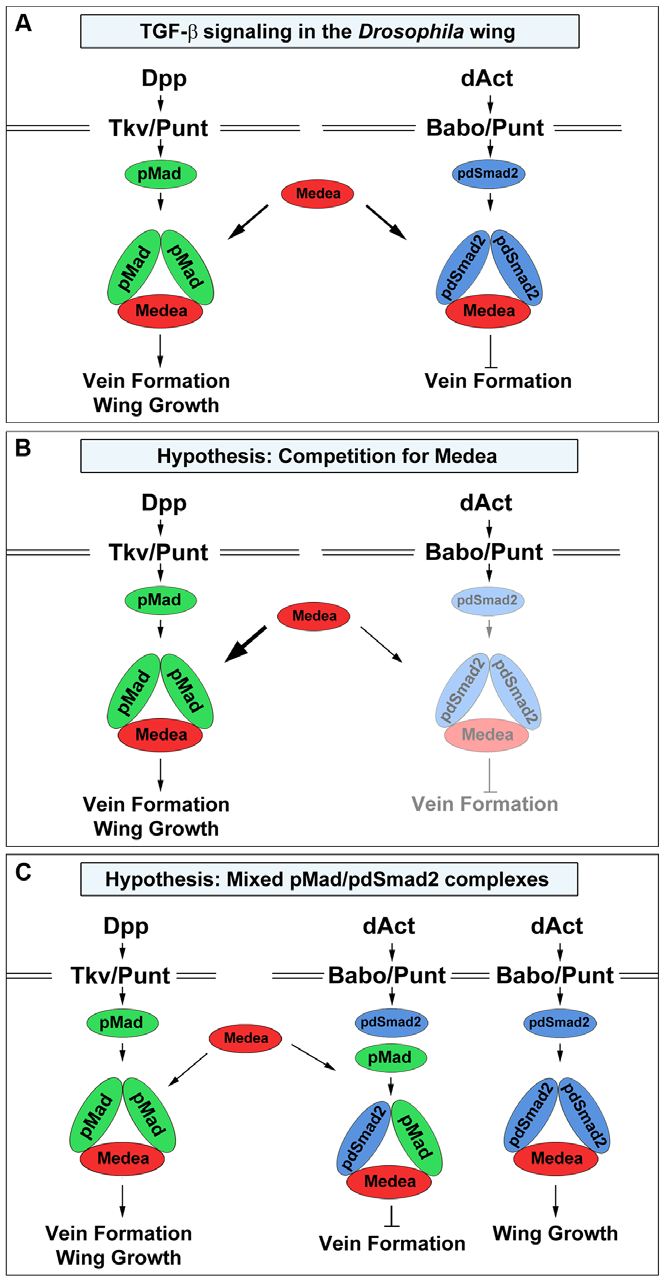
Hypothetical mechanisms for the opposing effects of dSmad2 on wing vein formation. (A) The Dpp signal is transduced by the receptors Thickveins and Punt that activate Mad via C-terminal phosphorylation. A heterotrimeric complex consisting of pMad and Medea is formed. This complex is translocated into the nucleus to initiate target gene expression for vein formation and wing growth. The *Drosophila* Activin (dAct) signal is transduced by the receptors Baboon and Punt; dSmad2 is phosphorylated and associates with Medea forming a heterotrimer. In the present study, we describe an inhibitory effect of dSmad2 signaling on vein formation that is mediated by an as yet unknown molecular mechanism. (B) The competition for Medea model places Medea as the limiting factor between Mad and dSmad2 signaling pathways. The phosphorylated transcription factors compete for association with rate-limiting amounts of Medea. Thus, when the level of dSmad2 is experimentally lowered, the Dpp/Mad branch receives an excess of Medea with which it can form heterotrimeric complexes. This leads to an elevated Mad/Medea response, which is manifested in the formation of extra vein tissue. (C) The mixed pMad/pdSmad2 complexes model is based on the finding by Gesualdi and Haerry [17] that the type I receptor Baboon is able to phosphorylate C-terminal serines of both dSmad2 and Mad. Mixed complexes composed of pMad, pdSmad2 and Medea may be responsible for inhibiting Mad-mediated vein formation. In parallel, the canonical pdSmad2/Medea complex could contribute to wing growth [17] or be involved in other unknown processes.

Figure 7A summarizes our findings on the function of dSmad2 in repressing vein formation in the *Drosophila* wing. In this diagram, both, pMad and pdSmad2 require Medea to form a trimeric complex. In a normal situation, pMad/Medea promotes vein formation and wing growth, while pdSmad2/Medea heterotrimers would act as inhibitors that fine-tune venation and growth.

One possible molecular explanation for the inhibitory effect of dSmad2 on Mad signaling is the competition for Medea, as depicted in Figure 7B. If Medea were a rate-limiting factor shared by the Mad and dSmad2 pathways, the knockdown of dSmad2 would free Medea to form complexes predominantly with pMad^cter^, transducing a stronger Mad signal that manifests itself by extra vein formation. The vertebrate homolog of Medea, Smad4, has been proposed to act as the rate-limiting step between Smad1 and Smad2 signal transduction in *Xenopus* embryos [20].

One reason for the limited availability of Medea may be the known instability of this protein. Recent work from the Piccolo group has demonstrated a cycle of monoubiquitination and deubiquitination of Smad4. Ubiquitinated Smad4 cannot interact with R-Smads in a trimeric complex, rendering signal transduction inactive. Subsequent deubiquitination reactivates Smad4 and makes it accessible for complex formation [50]. This cycle is essential for TGF-β signaling, since phosphorylated R-Smads are unable to signal in the absence of the deubiquinase FAM/USP9x (Fat facets in mouse/Ubiquitin-specific peptidase 9, X-linked). The *Drosophila* homolog of FAM, Fat Facets [51], was shown to have pro-Dpp activity when overexpressed in the *Drosophila* wing, suggesting that the regulation of Medea/Smad4 levels is critical and is conserved between flies and vertebrates.

An additional possibility is that Smad1/Mad and dSmad2 might compete for the type II receptors Punt or Wit which are common to both pathways. Evidence for ActR2 as the limiting factor in the switch between TGF-β/Nodal and BMP signaling has been recently obtained by Yamamoto and co-workers [27] during formation of distal visceral endoderm in the mouse embryo.

An alternative molecular mechanism, depicted in Figure 7C, is based on the ability of TGF-β receptors to phosphorylate both Smad2 and Smad1. Mixed Smad1/Smad2/Smad4 trimeric complexes have been shown in many normal and transformed cell lines of epithelial and endothelial origin [52]–[55]. In *Drosophila* S2 cells, dActivin and Baboon can induce the phosphorylation of Mad in addition to that of dSmad2 [17]. Smad1 phosphorylation by TGF-β receptor requires a combination of Smad1- and Smad2-specific type I TGF-β receptors in addition to the type II receptor, a higher ligand concentration, and starts later than that of Smad2/3 [55].

Several studies have reported different, in some cases, opposing transcriptional responses to activation of the canonical TGF-β/Smad2 pathway versus the alternative TGF-β/Smad1/Smad2 signal in normal and cancer cells [53]–[55]. In the model in Figure 7C, if dAct/Babo phosphorylated Mad in addition to dSmad2 in the developing wing, mixed trimeric complexes of pMad/pdSmad2/Medea might repress vein formation, hence opposing the vein-promoting function of Dpp/Mad. The ratios between “canonical” and “mixed” Smad complexes could fine-tune vein specification in the wing. Other potential mechanisms could be envisaged as well.

In conclusion, the present study in the *Drosophila* wing has revealed that an important function of dSmad2 in wing development is to antagonize Mad/Dpp signaling during vein formation. The molecular mechanism of this unexpected inhibitory activity of dSmad2 is as yet unknown, and several hypothetical possibilities have been discussed. Whether signaling in the opposite direction - an inhibitory effect of Mad on dSmad2 signaling – occurs in *Drosophila* remains to be investigated. In the vertebrates, there is much evidence showing that the opposition between the BMP and TGF-β pathways is mutual [20], [26], [27], suggesting that this could be a promising area for future research.

## Materials and Methods

### *Drosophila* Transgenic Constructs

The dSmad2 RNAi strain used in most of this study (Transformant ID 14609) was obtained from the Vienna Drosophila RNAi Center (VDRC). It originates from the “GD” P-element library of UAS-RNAi constructs cloned into pMF3, a modification of the pUAST vector [36]. This dSmad2 RNAi is targeted against nucleotides 652–1049 of the dSmad2 coding sequence, which corresponds to parts of both the linker region and the MH2 domain of dSmad2, and was inserted on chromosome 3. A second dSmad2 RNAi strain (Transformant ID 105687, targeted against nucleotides 149–559) from the “KK” phiC31 library, inserted on chromosome 2, was used as a specificity control. Baboon RNAi flies were also obtained from Vienna (Transformant ID 3825, targeting 171 nucleotides), inserted in chromosome 3. The main Medea RNAi strain used was: Transformant ID 106767 from the VDRC KK library, inserted on chromosome 2 and targeted against nucleotides 1434–1883. An independent Medea RNAi line (Transformant ID 19688 from the GD library, inserted on chromosome 3 and targeted against nucleotides 258–551) was used as a specificity control and caused identical phenotypes (data not shown). The Mad RNAi strain used here was generated as described by Eivers et al. [6]. Full-length N-terminal Myc-tagged hSMAD3 (kind gift from Elizabeth Robertson) was first cloned into the *Xenopus* expression vector pCS2+ using *Bam*HI and *Xho*I restriction sites and then subcloned into the pUAST vector [56] using *Not*I and *Xho*I sites, and stable transgenic fly lines were generated by the Bestgene company (Chino Hills, CA.).

### Fly Strains

Single RNAi strains used were: 1) yw;Bl/CyO;dSmad2 RNAi/TM6b, 2) yw;Medea RNAi/CyO;TM2/TM6b, 3) yw;Mad RNAi/CyO;TM2/TM6b. The hSMAD3 wild-type strain used was yw;hSMAD3/Bl;TM2/TM6b. Mad wild-type was yw;Mad/CyO;TM2/TM6b [6]. The Strain for the RNAi rescue experiment was: yw;hSMAD3/CyO;dSmad2 RNAi/TM6b. Strains for epistasis experiments were yw;Mad RNAi/Bl;dSmad2 RNAi/TM6b, and yw;Medea RNAi/CyO;dSmad2 RNAi/TM6b. Gal4 drivers (Bloomington stock number in parentheses) were as follows: MS1096-Gal4 (#8696), Scalloped-Gal4 (#8609), and A9-Gal4. Another strain used in this study was UAS-Dpp (#1486). Flies were grown at 25°C unless stated differently.

### Clonal Analysis

For random “flp-out” clones [39] we crossed females of the genotype yw;Act>y^+^>Gal4;UAS-GFP (kind gift of K. Pappu) to the following males: ywhsflp;dSmad2 RNAi/dSmad2 RNAi. Single heat shocks were administered to embryos 24 hrs after egg laying for 60 min at 37°C. After heat-shock, larvae were grown at 25°C for recovery and further development.

### Wing Disc Preparation

Wing discs were dissected from third instar larvae in cold 0.02% Triton X-100 PBS (PBST) solution. Discs were fixed in 4% formaldehyde for 30 minutes on ice, rinsed in PBST, and placed in DAPI-containing Vectashield (Vector) before mounting on glass slides.

### Pupal Wing Preparation and Immunostaining

In general, paupal wing stainings followed the procedures described in the supplementary methods of Yan et al. [41]. Briefly, late third instar larvae were selected and left to develop for 25 hrs after puparium formation (APF) at 25°C. Pupae were dissected out of the puparium case, fixed in 4% formaldehyde in PBS (either overnight at 4°C or 1 hr at room temperature), and rinsed three times in 0.3% PBST. Pupal wings were dissected from the pupae in PBST, and wing cuticles manually removed with care. The rest of the protocol was carried out as described above for wing disc immunostainings, using 0.2% BSA in 0.3% PBST for blocking and antibody dilutions. Antibodies used were anti-pMad^cter^ (1∶200, generous gift from C.-H. Heldin), followed by goat anti-rabbit Cy3-conjugated secondary antibody (1∶1000, Jackson Labs). Wing discs and pupal wings were photographed using a Zeiss Axio Imager.Z1 microscope equipped with Zeiss ApoTome oscillating grating in the epifluorescence beam, which significantly reduces out of focus stray light.

### Mounting of Adult Wings

Wings were removed from female adult flies and dehydrated in 100% ethanol for 5 mins. The wings were placed on a microscope slide, and ethanol allowed to evaporate. A small drop of Canada balsam was dropped onto the wing and a glass coverslip placed on top. Preparations were flattened at 60°C with a 10 g weight on top. Wings were photographed with a Zeiss Axiophot microscope using a Leica DC500 or a Zeiss AxioCam HRc camera. In general, 10–20 images at different focal planes were integrated using the AxioVision 4.8 Zeiss software.

## Supporting Information

Figure S1 Two non-overlapping dSmad2 RNAi strains cause extra vein formation and smaller wings. (A) Schematic representation of the domain structure of dSmad2 indicating the location of the two VDRC dSmad2 RNAi strains (MH, Mad Homology). (B) RT-PCR of dSmad2 depleted third instar imaginal wing discs showing a 70% decrease in mRNA expression compared to controls. dSmad2 RNAi was driven using scalloped-Gal4; this experiment shows that dSmad2 is an effective loss-of-function reagent. (C and D) dSmad2 RNAi KK105687, driven with MS1096-Gal4 throughout the wing blade, leads to ectopic vein formation near L5 and smaller wings (compare to Figure 1B). (E and F) Overlays of dSmad2 RNAi driven wings compared to wild-type wings. Both dSmad2 RNAi strains reduce the size of the wing blade. The two independent dSmad2 RNAi strains obtained from VDRC produce similar phenotypes in the wing: ectopic vein formation and a moderate decrease in wing size.
(3.75 MB TIF)

Figure S2 dSmad2 RNAi leads to formation of ectopic bristles on the wing blade. (A–C) dSmad2 RNAi (GD14609 line) expressed throughout the wing blade using the A9-Gal4 driver causes formation of ectopic sensory bristles (arrows) in the anterior and distal portion of the wing blade at 29°C. (D–F) The KK105687 dSmad2 RNAi line driven by MS1096-Gal4 leads to similar ectopic bristle formation when grown at 25°C. In most cases, a sensory bristle is formed at the end of an extra vein that branches from the distal part of L2. Sensory bristle formation in the anterior wing margin requires Wingless (Wg) signaling [57], and generation of ectopic sensory bristles on the wing blade is seen in clones of activated Wg, such as Shaggy (Sgg; zeste white 3, zw3; Glycogen Synthase Kinase 3, GSK3) null clones [58]. In a recent study on Wg signal integration at the level of Mad, it was shown that overexpression of Mad mutants with phosphorylation-resistant GSK3 sites in the linker region of the protein (or “Mad GSK3 Mutants”) resulted in hyperactive forms of Mad that caused ectopic bristle formation, mimicking activation of Wg signaling. Conversely, Mad knockdown by RNAi phenocopied Wg loss-of-functions. It was concluded that the Wg-like phenotypes achieved by Mad loss- or gain-of-function are mediated through the Wg-regulated phosphorylation of Mad by GSK3 in the linker region [6]. Based on these observations, we propose that depletion of dSmad2 by RNAi causes elevated Mad signaling, comparable to that attained by Mad GSK3 mutant overexpression. All wings were from female flies except for E and F. The frequency of the ectopic bristle phenotype was higher in male flies due to the higher expression of MS1096-Gal4 in males.
(3.16 MB TIF)
