# Supplementary figures and images for: Drosophila Smad2 Opposes Mad Signaling during Wing Vein Development
Source: PLoS One. 2010 Apr 28;5(4):e10383. doi: 10.1371/journal.pone.0010383 (PMC2860994; doi:10.1371/journal.pone.0010383)

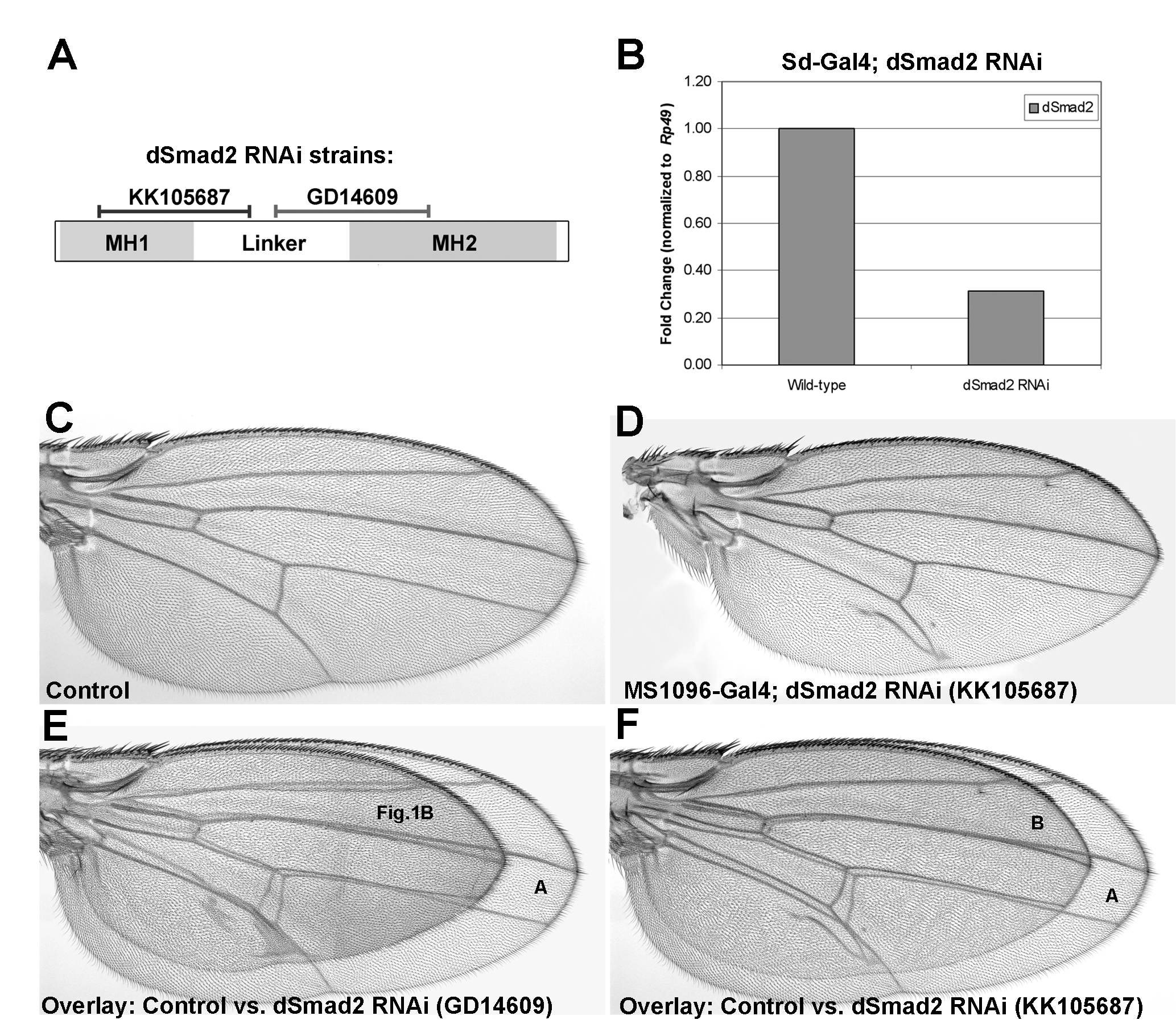

Supplement: Figure S1 — Two non-overlapping dSmad2 RNAi strains cause extra vein formation and smaller wings. (A) Schematic representation of the domain structure of dSmad2 indicating the location of the two VDRC dSmad2 RNAi strains (MH, Mad Homology). (B) RT-PCR of dSmad2 depleted third instar imaginal wing discs showing a 70% decrease in mRNA expression compared to controls. dSmad2 RNAi was driven using scalloped-Gal4; this experiment shows that dSmad2 is an effective loss-of-function reagent. (C and D) dSmad2 RNAi KK105687, driven with MS1096-Gal4 throughout the wing blade, leads to ectopic vein formation near L5 and smaller wings (compare to Figure 1B). (E and F) Overlays of dSmad2 RNAi driven wings compared to wild-type wings. Both dSmad2 RNAi strains reduce the size of the wing blade. The two independent dSmad2 RNAi strains obtained from VDRC produce similar phenotypes in the wing: ectopic vein formation and a moderate decrease in wing size. (3.75 MB TIF) [file pone.0010383.s001.tif]

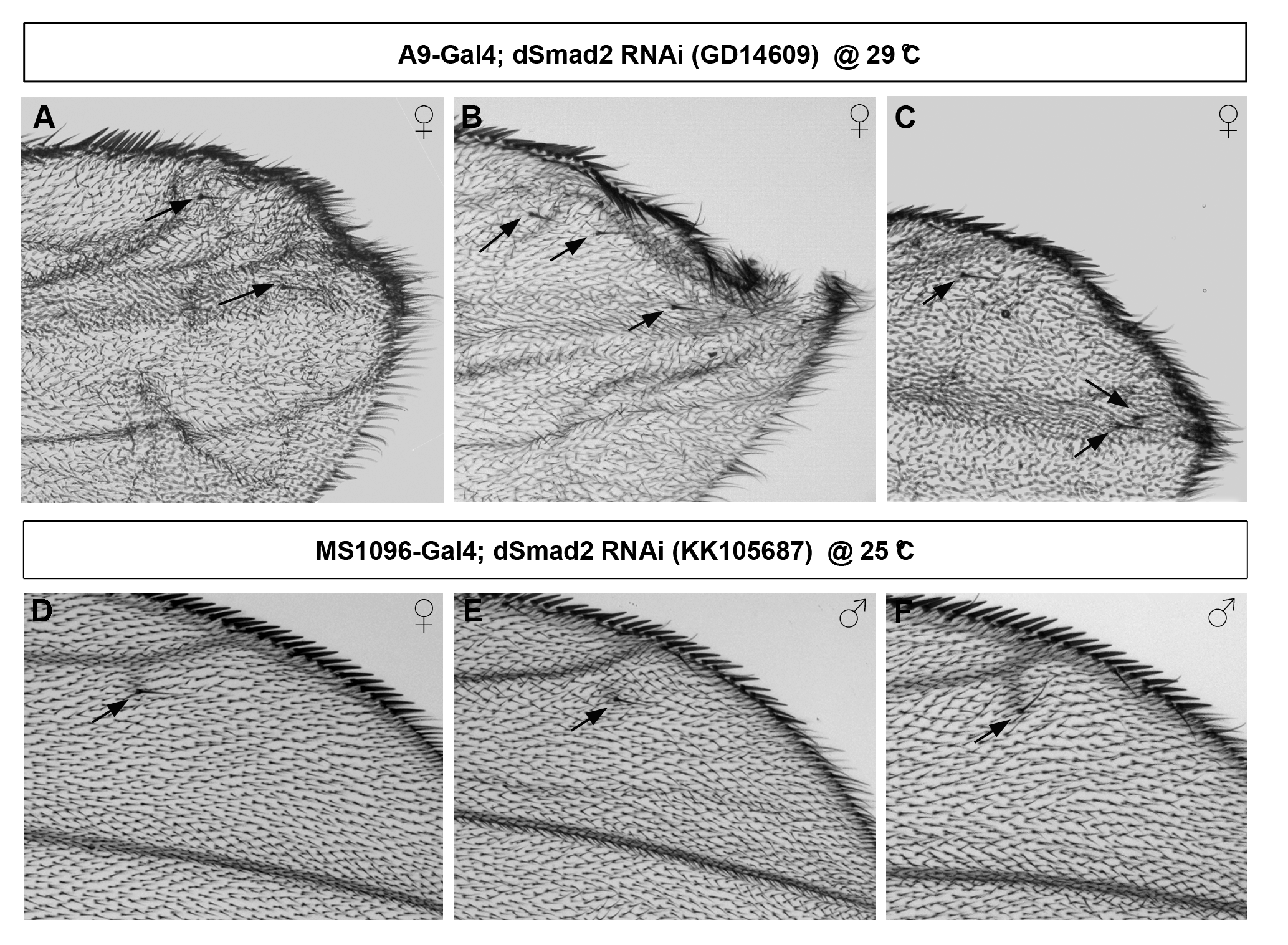

Supplement: Figure S2 — dSmad2 RNAi leads to formation of ectopic bristles on the wing blade. (A–C) dSmad2 RNAi (GD14609 line) expressed throughout the wing blade using the A9-Gal4 driver causes formation of ectopic sensory bristles (arrows) in the anterior and distal portion of the wing blade at 29°C. (D–F) The KK105687 dSmad2 RNAi line driven by MS1096-Gal4 leads to similar ectopic bristle formation when grown at 25°C. In most cases, a sensory bristle is formed at the end of an extra vein that branches from the distal part of L2. Sensory bristle formation in the anterior wing margin requires Wingless (Wg) signaling [57], and generation of ectopic sensory bristles on the wing blade is seen in clones of activated Wg, such as Shaggy (Sgg; zeste white 3, zw3; Glycogen Synthase Kinase 3, GSK3) null clones [58]. In a recent study on Wg signal integration at the level of Mad, it was shown that overexpression of Mad mutants with phosphorylation-resistant GSK3 sites in the linker region of the protein (or “Mad GSK3 Mutants”) resulted in hyperactive forms of Mad that caused ectopic bristle formation, mimicking activation of Wg signaling. Conversely, Mad knockdown by RNAi phenocopied Wg loss-of-functions. It was concluded that the Wg-like phenotypes achieved by Mad loss- or gain-of-function are mediated through the Wg-regulated phosphorylation of Mad by GSK3 in the linker region [6]. Based on these observations, we propose that depletion of dSmad2 by RNAi causes elevated Mad signaling, comparable to that attained by Mad GSK3 mutant overexpression. All wings were from female flies except for E and F. The frequency of the ectopic bristle phenotype was higher in male flies due to the higher expression of MS1096-Gal4 in males. (3.16 MB TIF) [file pone.0010383.s002.tif]
